# Supplementary material for: Heat stable and intrinsically sterile liquid protein formulations
Source: Nat Commun. 2024 Dec 30;15:10897. doi: 10.1038/s41467-024-55304-9 (PMC11685576; doi:10.1038/s41467-024-55304-9)
Supplement: Supplementary file 1 — Supplementary Information [file 41467_2024_55304_MOESM1_ESM.pdf]

Supplementary Information

Heat Stable and Intrinsically Sterile Liquid Protein Formulations

Atip Lawanprasert, Harminder Singh, Sopida Pimcharoen, Mariangely González Vargas, Arshiya Dewan, Girish S. Kirimanjeswara, and Scott H. Medina

Content **Page Number**

Materials **S1**

Supplementary Fig. 1. Representative DSC heat capacity protein profiles. **S2**

Supplementary Fig. 2. ^{1}H-NMR spectra of PFNA:BSA complex **S3**

Supplementary Fig. 3. ^{19}F-NMR spectra of PFNA:BSA complex **S4**

Supplementary Fig. 4. FTIR Amide protein spectra after PFNA interaction **S5**

Supplementary Fig. 5. FTIR C–F spectra after PFNA-protein interaction **S6**

Supplementary Fig. 6. Protein-dependent ^{19}F NMR shift of PFOc spectral peaks **S7**

Supplementary Fig. 7. PFOc-BSA extended bacterial contamination **S8**

Supplementary Fig. 8. Calibration curve for quantification of β-gal serum concentration **S9**

Supplementary Fig. 9. Calculated β-gal pharmacokinetic parameters **S10**

Supplementary Fig. 10. Time-dependent serum proteolysis of β-gal **S11**

Supplementary Fig. 11. Full size figure of serologic toxicology results **S12**

Table S1: Tabulated serological toxicity results **S13**

Supplementary Fig. 12. H&E tissue sections (40X magnification) **S14**

Materials

n-Perfluorooctane (PFOc) solvent was purchased from Oakwood Chemical (Estill, NC). Perfluorononanoic acid (PFNA) was purchased from Alfa Aesar (Haverhill, MA). 1X Phosphate Buffer Saline (PBS) was purchased from Corning (Corning, NY). Coomassie Protein Assay Reagent 1X, Bovine Serum Albumin (BSA) and o-Nitrophenyl-β-D-galactopyranoside (ONPG) were purchased from was purchased from Fisher Scientific (Hampton, NH). Human Hemoglobin Lyophilized powder, β-Galactosidase from E. *coli* Grade VI (MW 465,000 Da, >250 U/mg), Human Serum IgG, Trypsin from Bovine Pancreas Type I, Proteinase K from T. *album*, 4-methylumbelliferyl β-D-galactopyranoside (μ-Gal), Nα-Benzoyl-DL-arginine 4-nitroanilide hydrochloride (BAEE) were purchased from Sigma-Aldrich (St. Louise, MO). 50% v/v Hydrochloric acid (HCl) was purchased from Aqua solution (Jasper, GA). 100% Bleach solution was acquired from Clorox™ (Oakland, CA). Green Fluorescent Protein (GFP) was a generous gift from Dr. Joel Schneider of the National Cancer Institute (Bethesda, MD).

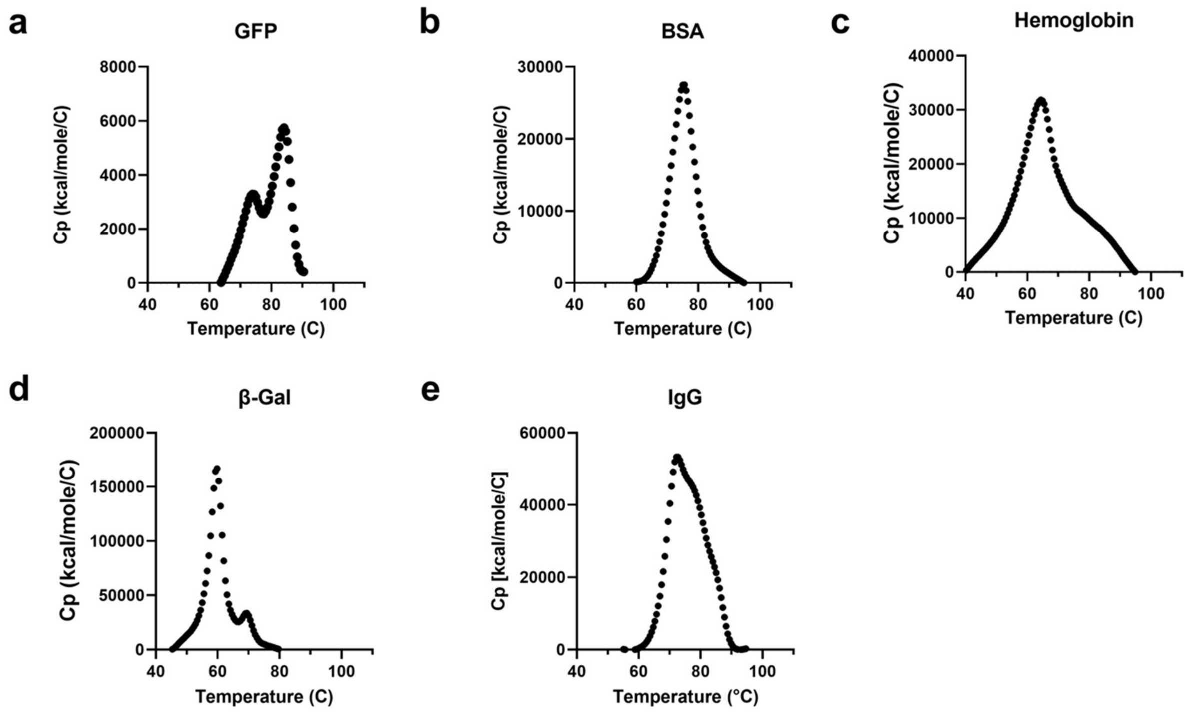

Supplementary Fig. 1. Representative DSC heat capacity protein profiles. (**a**) GFP, (**b**) BSA, (**c**), Hemoglobin, (**d**) β-Gal, and (**e**) IgG in saline. Experiment was repeated three times with similar results.

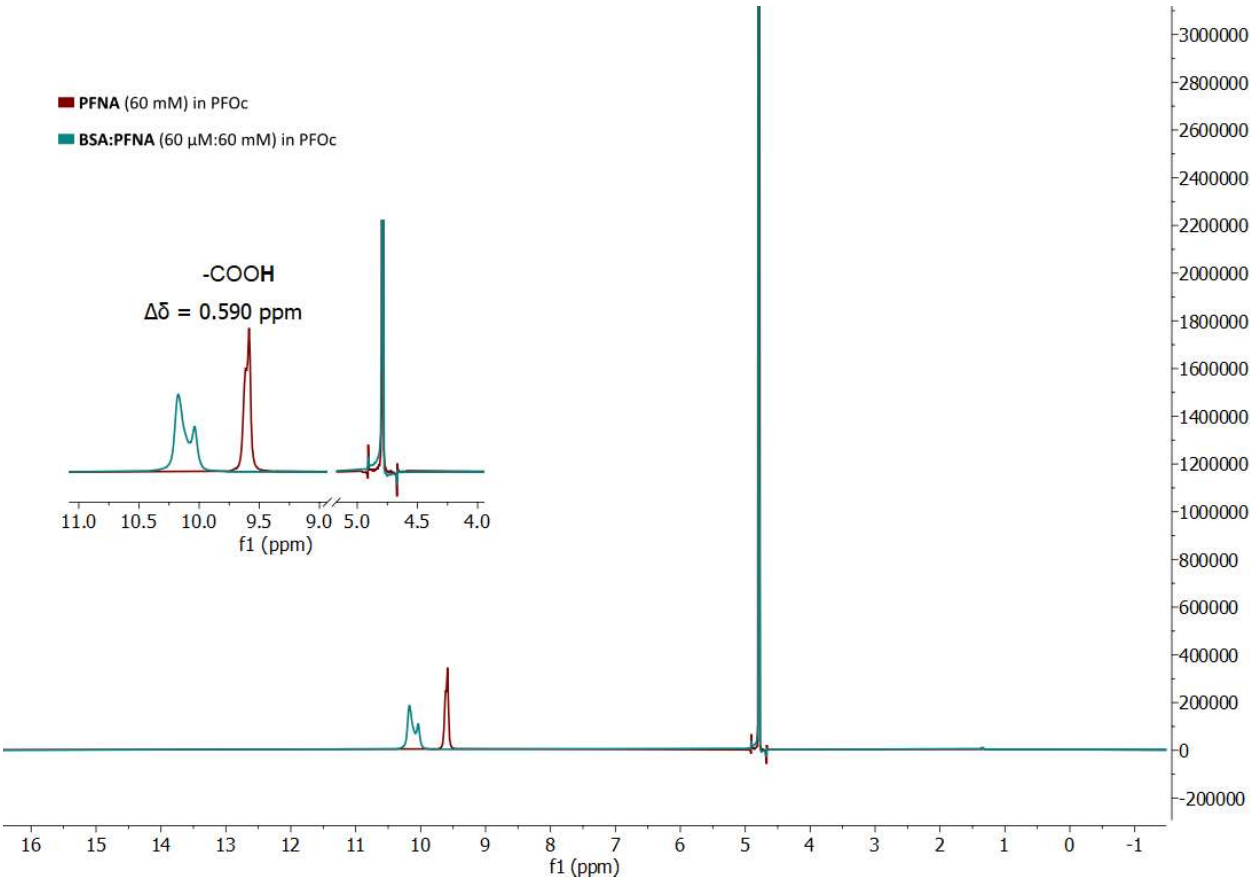

Supplementary Fig. 2. ¹H-NMR spectra of PFNA:BSA complex. PFNA (red) or BSA:PFNA complex (blue, 1:1000 molar ratio). Experiment was repeated three times with similar results.

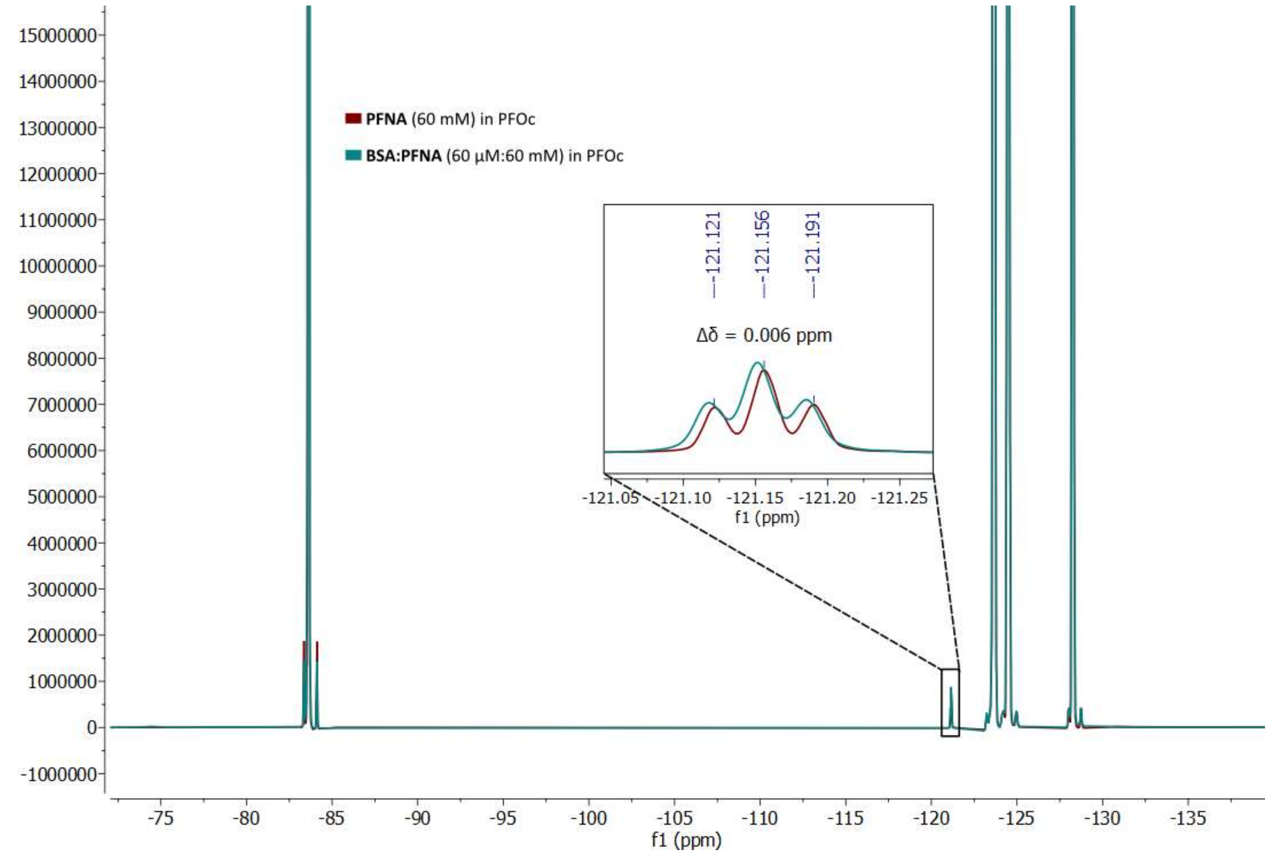

Supplementary Fig. 3. ^{19}F-NMR spectra of PFNA:BSA complex. PFNA (red) or BSA:PFNA complex (blue, 1:1000 molar ratio). Experiment was repeated three times with similar results.

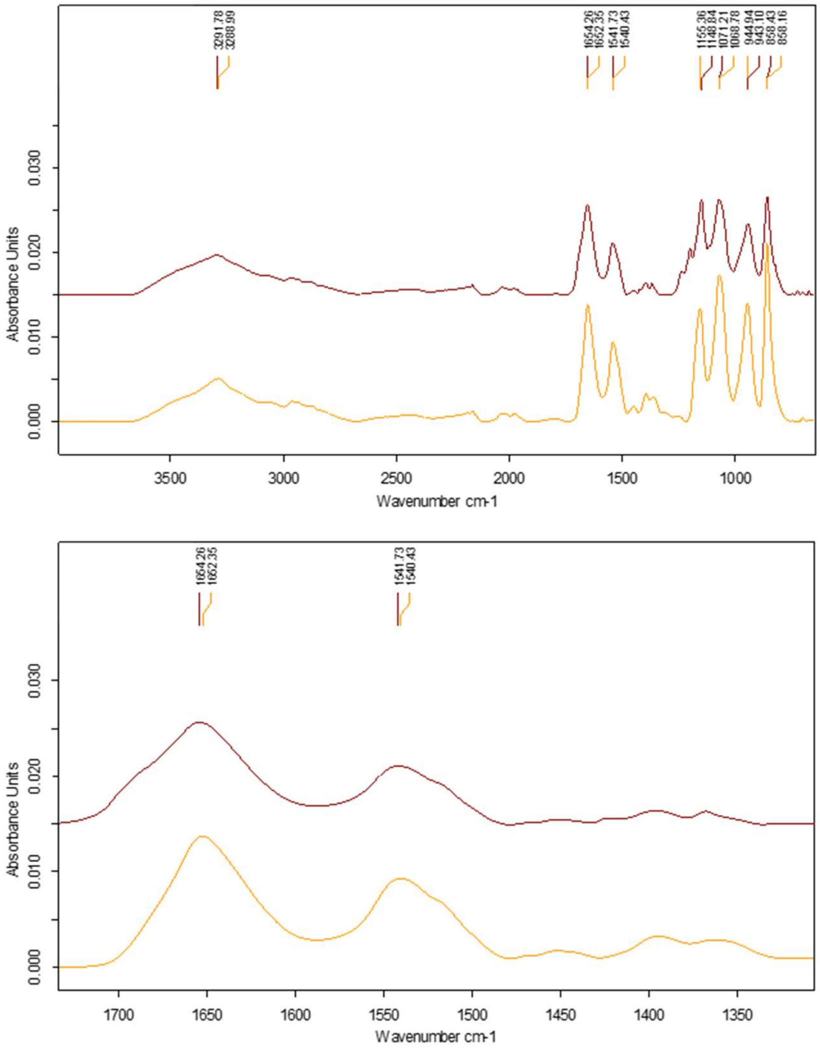

Supplementary Fig. 4. FTIR Amide protein spectra after PFNA interaction. Representative spectra are shown in (top) full or (bottom) magnified between 1750 – 1300 cm^{-1}. Spectra were taken from lyophilized (orange) free BSA and (brown) PFNA-treated BSA samples. Experiment was repeated three times with similar results.

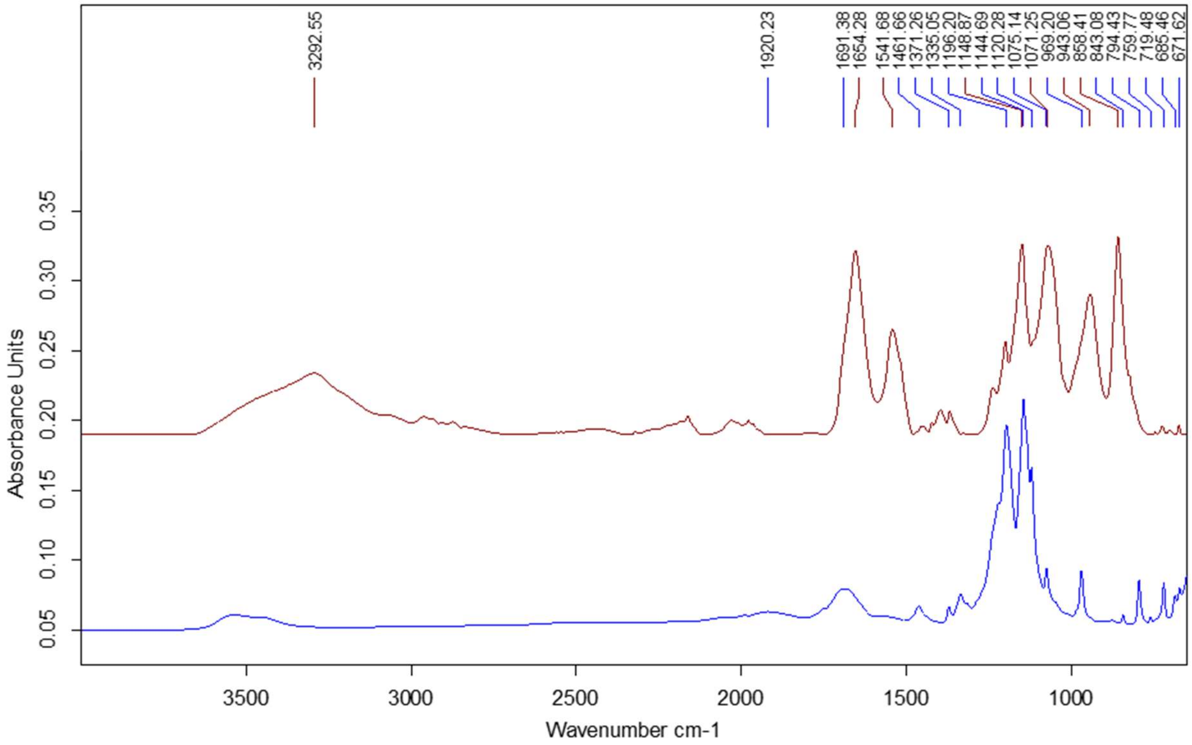

Supplementary Fig. 5. FTIR C–F spectra after PFNA-protein interaction. Representative spectra were acquired from (blue) PFNA or (brown) lyophilized BSA-treated PFNA samples. Experiment was repeated three times with similar results.

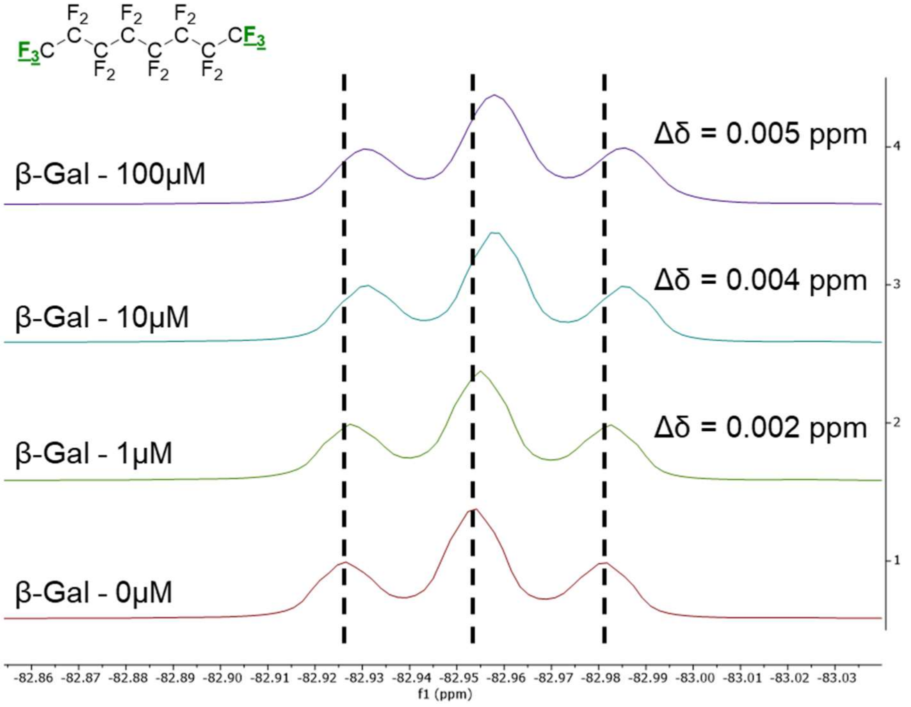

Supplementary Fig. 6. Protein-dependent ^{19}F NMR shift of PFOc spectral peaks. Stacked representative ^{19}F NMR spectra demonstrating PFOc's -C**<u>F</u>** chemical shift (Δδ) as a function of increasing concentration of PFNA-dispersed β-Gal (0 – 100μM). Dashed lines are shown to aid in visualization of peak shift; PFNA concentration was kept constant at 1mM for all conditions. Experiment was repeated three times with similar results.

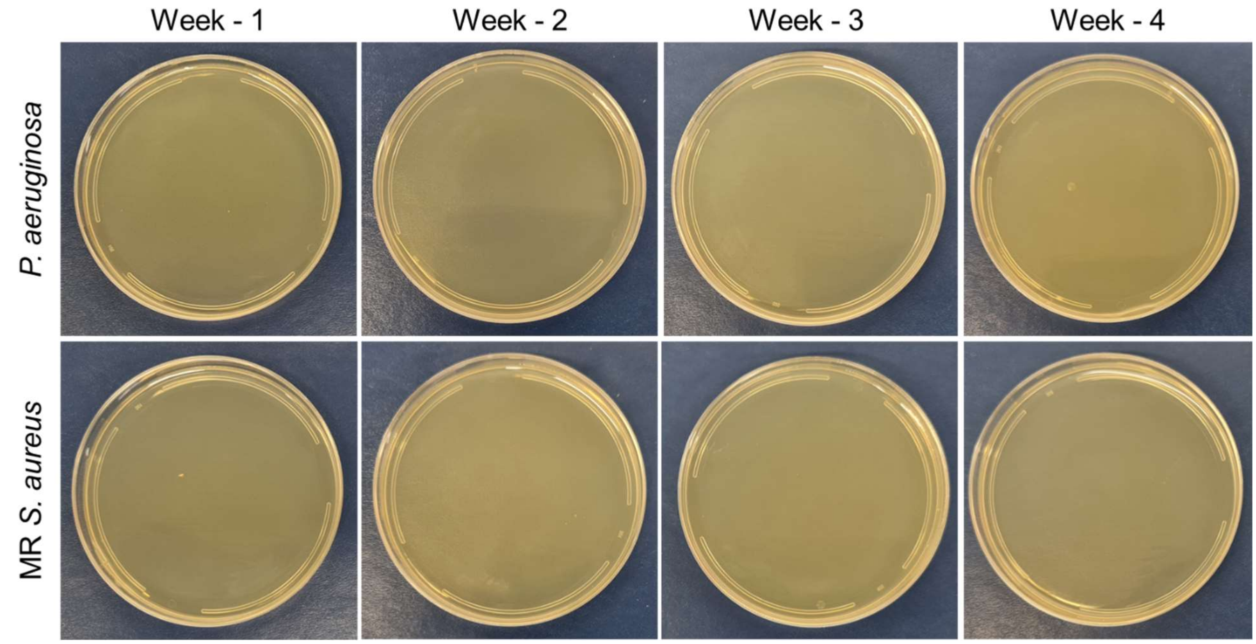

Supplementary Fig. 7. PFOc-BSA extended bacterial contamination. Representative optical images of agar plates after addition of PFNA-BSA in PFOc samples contaminated with (*top row*) *P. aeruginosa* or (*bottom row*) Methicillin resistant *S. aureus* and allowed to culture for 1 – 4 weeks. Experiment was repeated three times with similar results.

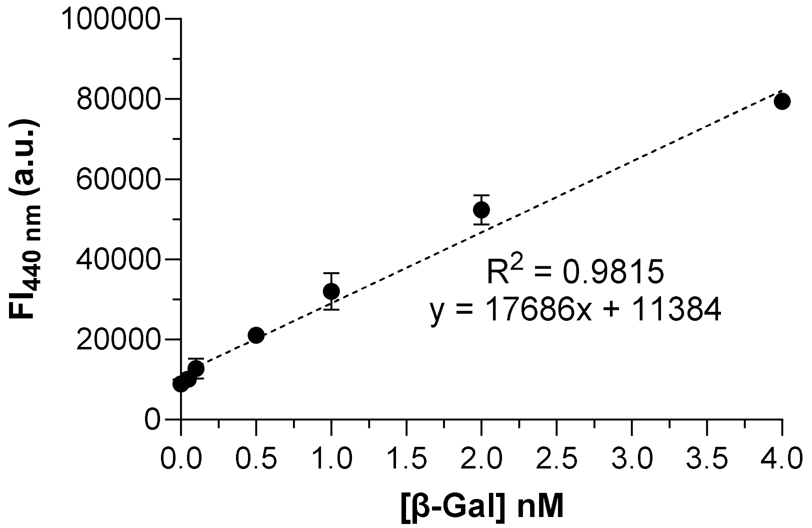

Supplementary Fig. 8. Calibration curve for quantification of β-gal serum concentration. Calibration curve relating the fluorescence intensity (FI_{440nm}) of the converted β-gal substrate, 4-Methylumbelliferyl-α-D-galactopyranoside, vs. protein concentration of n = 3 technical replicates.

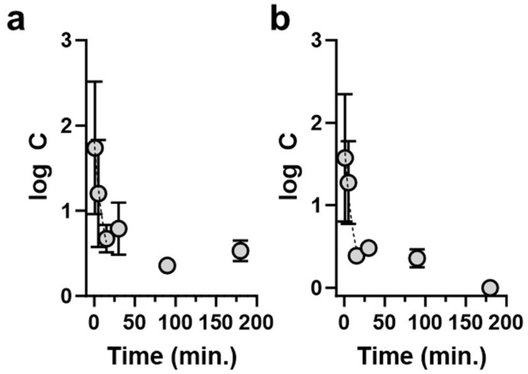

c

Parameter	β-Gal$_{Saline}$	β-Gal$_{Ext.\ PFOc}$	P value
**D** [mg/kg]	1.55 ± 0	1.55 ± 0	-
**C_0** [mg/L]	45.97 ± 65.58	35.35 ± 47.53	0.777
**$t_{1/2}$** [min]	7.12 ± 3.83	5.80 ± 2.03	0.517
**V_d** [mL/kg]	104.75 ± 96.55	99.11 ± 64.74	0.916
**k_e** [min^{-1}]	0.12 ± 0.07	0.14 ± 0.08	0.739
**Cl** [mL/min·kg]	8.65 ± 4.66	10.77 ± 5.56	0.531
**AUC** [mg·hr/L]	14.45 ± 3.22	10.06 ± 2.90	0.053

Supplementary Fig. 9. Calculated β-gal pharmacokinetic parameters. (**a**,**b**) Log concentration of β-Gal in mouse serum versus sampling time for (a) β-Gal$_{Saline}$ or (b) β-Gal$_{Ext.\ PFOc}$. Dashed line represents linear regime used for pharmacokinetic calculations. (**c**) Pharmacokinetic parameters for β-Gal$_{Saline}$ or β-Gal$_{Ext.\ PFOc}$ formulations, shown as mean value ± standard deviation. P values determined using Student's t-test of n = 4 technical replicates.

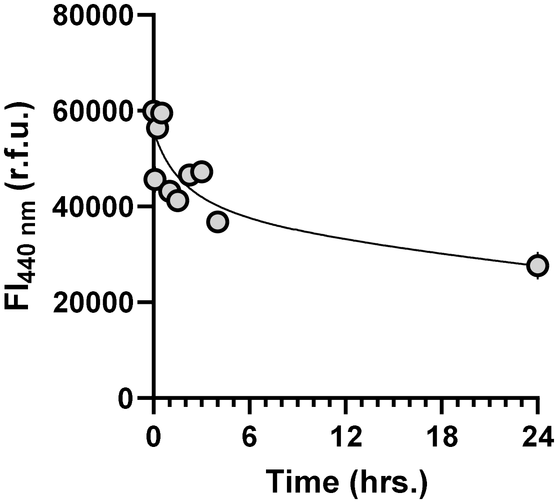

Supplementary Fig. 10. Time-dependent serum proteolysis of β-gal. Fluorescence intensity (FI_{440nm}, relative fluorescence units) of the converted β-gal substrate, 4-Methylumbelliferyl-α-D-galactopyranoside, 5 minutes after its treatment with 1 μM β-Gal pre-incubated in mouse serum at varying time points. Results shown as mean value ± standard deviation of n = 3 technical replicates.

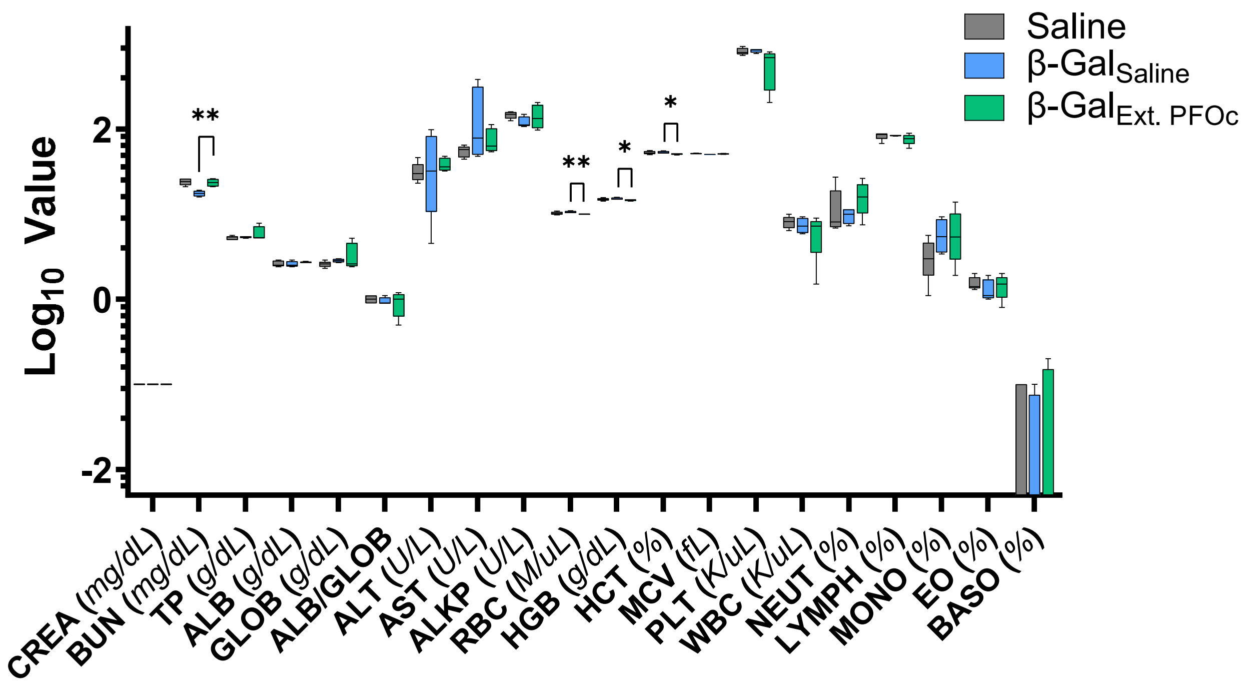

Supplementary Fig. 11. Full size figure of serologic toxicology results. Full size figure showing serologic toxicology results from C57BL/6J mice 24 hours after administration of saline (control), β-Gal$_{Saline}$ or β-Gal$_{Ext. PFOc}$. Data shown as box and whisker plot ± s.d. of n = 4 technical replicates. Statistical significance determined using Student's t-test and represented as * $p < 0.05$; all other comparisons were found not to be significant ($p > 0.05$).

Table S1: **Tabulated serological toxicity results**

Marker	Saline		β-Gal$_{Saline}$		β-Gal$_{Ext. PFOc}$	
	Average	*Std. Dev.*	*Average*	*Std. Dev.*	*Average*	*Std. Dev.*
CREA (*mg/dL*)	0.10	0.00	0.10	0.00	0.10	0.00
BUN (*mg/dL*)	24.00	2.12	24.00	2.65	17.50	1.29
TP (*g/dL*)	5.28	0.27	6.10	1.47	5.40	0.14
ALB (*g/dL*)	2.62	0.22	2.73	0.06	2.58	0.22
GLOB (*g/dL*)	2.58	0.22	3.40	1.56	2.85	0.13
ALB/GLOB	1.00	0.10	0.90	0.36	0.95	0.10
ALT (*U/L*)	31.40	8.73	38.00	8.72	41.63	39.83
AST (*U/L*)	54.60	8.20	79.67	29.02	146.75	159.15
ALKP (*U/L*)	145.80	13.88	139.67	57.46	120.00	19.48
RBC (*M/uL*)	10.20	0.41	9.94	0.13	10.54	0.27
HGB (*g/dL*)	14.90	0.56	14.53	0.25	15.15	0.47
HCT (%)	52.66	2.19	50.65	0.95	53.15	1.74
MCV (*fL*)	51.62	0.33	50.98	0.54	50.43	0.39
PLT (*K/uL*)	816.20	77.85	526.25	286.39	834.25	43.87
WBC (*K/uL*)	8.06	1.32	5.44	2.76	7.42	1.60
NEUT (%)	11.98	8.67	13.73	4.74	9.65	1.99
LYMPH (%)	83.24	8.78	80.15	6.18	83.15	0.58
MONO (%)	3.20	1.63	4.45	1.98	5.90	2.70
EO (%)	1.54	0.28	1.60	0.29	1.28	0.42
BASO (%)	0.04	0.05	0.08	0.10	0.03	0.05

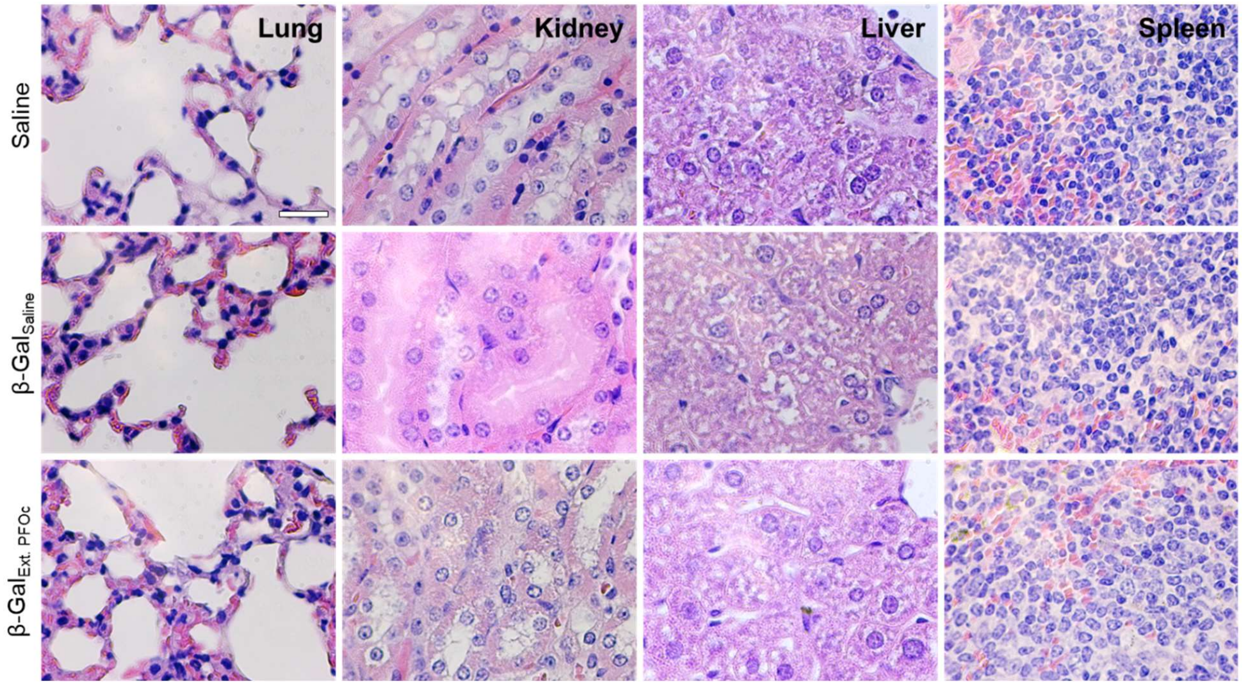

Supplementary Fig. 12. H&E tissue sections (40X magnification). Representative histopathological analysis of organs. 40X magnification of H&E stained lung, kidney, liver and spleen tissue sections from mice treated with saline (control), β-Gal$_{Saline}$ or β-Gal$_{Ext. PFOc}$. Scale bar = 25 μm. Each treatment group consisted of n = 4 mice and analysis was performed on four random fields at 40X for each organ and analyzed in a blinded manner. Imaging was repeated four times with similar results.
